# Efficacy of Interceptor G2, Royal Guard and PermaNet 3.0 against pyrethroid-resistant *Anopheles gambiae* s.l. from Za-Kpota, southern Benin: an experimental hut trial

**DOI:** 10.1186/s13071-024-06372-9

**Published:** 2024-07-11

**Authors:** Pierre Marie Sovegnon, Romaric Akoton, Isaac J. Stopard, Thomas S. Churcher, Philip J. McCall, Hilary Ranson, Geraldine M. Foster, Luc Salako Djogbénou

**Affiliations:** 1 Tropical Infectious Diseases Research Centre (TIDRC), University of Abomey Calavi, Cotonou, Benin; 2 Department of Vector Biology, Liverpool School of Tropical Medicine, Pembroke Place, Liverpool, UK; 3 MRC Centre for Global Infectious Disease Analysis, School of Public Health, Faculty of Medicine, Imperial College London, London, UK

**Keywords:** *Anopheles gambiae*, Pyrethroid resistance, Insecticide-treated nets, Sublethal effect, PBO, Dual-active-ingredient insecticidal nets

## Abstract

**Background:**

The widespread use of insecticide-treated nets (ITNs) has significantly contributed to the reduction in malaria cases and deaths observed across Africa. Unfortunately, this control strategy is threatened by the rapid spread of pyrethroid resistance in malaria vectors. Dual-active-ingredient insecticidal nets are now available to mitigate the impact of pyrethroid resistance. To facilitate evidence-based decisions regarding product selection in specific use settings, data are needed on the efficacy of these different nets against local mosquito populations.

**Methods:**

Two experimental hut trials were performed in Za-Kpota, southern Benin in 2021 to evaluate the performance of Interceptor G2 (BASF), Royal Guard (Disease Control Technologies) and PermaNet 3.0 (Vestergaard Frandsen), all dual-active-ingredient bednets, in comparison to untreated or standard pyrethroid-treated bednets, against free-flying wild *Anopheles gambiae* mosquitoes. The performance of some of these next-generation nets was compared to the same type of nets that have been in use for up to 2 years. Mosquitoes collected in the huts were followed up after exposure to assess the sublethal effects of treatments on certain life-history traits.

**Results:**

The predominant species in the study site was *Anopheles gambiae* sensu stricto (*An. gambiae* s.s.). Both *Anopheles coluzzii* and *An. gambiae* s.s. were resistant to pyrethroids (deltamethrin susceptibility was restored by piperonyl butoxide pre-exposure). In the experimental hut trials, the highest blood-feeding inhibition (5.56%) was recorded for the Royal Guard net, relative to the standard PermaNet 2.0 net (44.44% inhibition). The highest 72-h mortality rate (90.11%) was recorded for the Interceptor G2 net compared to the PermaNet 2.0 net (56.04%). After exposure, the risk of death of *An. gambiae* sensu lato (*An. gambiae* s.l.) was 6.5-fold higher with the Interceptor G2 net and 4.4-fold higher with the PermaNet 3.0 net compared to the respective untreated net. Lower mosquito mortality was recorded with an aged Interceptor G2 net compared to a new Interceptor G2 net. Oviposition rates were lower in mosquitoes collected from huts containing ITNs compared to those of untreated controls. None of the mosquitoes collected from huts equipped with Royal Guard nets laid any eggs.

**Conclusions:**

The Royal Guard and Interceptor G2 nets showed a potential to significantly improve the control of malaria-transmitting vectors. However, the PermaNet 3.0 net remains effective in pyrethroid-resistant areas.

**Graphical Abstract:**

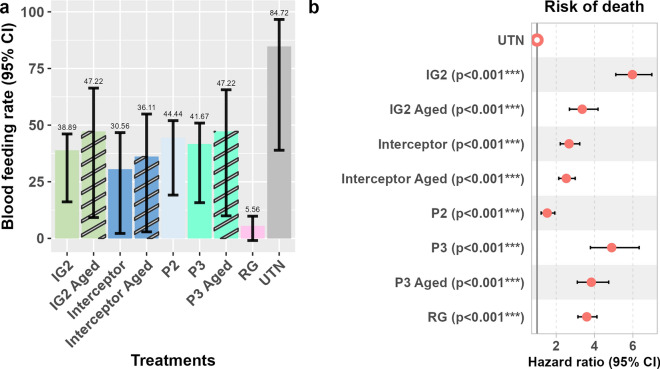

## Background

Insecticide-treated nets (ITNs) remain the key preventive tool for sustainable malaria control in endemic communities [1, 2]. Historically, all ITNs contained a single class of pyrethroid insecticide. However, the rapid increase in resistance to this class of insecticide in African malaria vectors [3] has driven the development and deployment of new types of ITNs that contain a second active ingredient in addition to the pyrethroid [4], referred to as dual-active-ingredient insecticidal nets. ITNs containing both pyrethroids and piperonyl butoxide (PBO; an insecticide synergist that primarily acts by inhibiting the action of oxidase enzymes in mosquito vectors) have been shown to be effective against pyrethroid-resistant *Anopheles gambiae* sensu lato (*An. gambiae* s.l.) and *Anopheles funestus* mosquitoes [5]. Recent large cluster randomised trials in Uganda [6] and Tanzania [7] showed that the pyrethroid-PBO nets Olyset Plus (Sumitomo Chemical, Tokyo, Japan) and PermaNet 3.0 (Vestergaard Frandsen, Lausanne, Switzerland) confer better protection against malaria than pyrethroid-only nets, leading the WHO to recommend pyrethroid-PBO nets in areas where the main malaria vectors exhibit intermediate levels of resistance mediated by the monooxygenase-based pyrethroid resistance mechanism [4]. However, there is still uncertainty regarding whether pyrethroid-PBO ITNs will perform better than pyrethroid-only ITNs against mosquito populations with extremely high levels of pyrethroid resistance or when other mechanisms, such as glutathione* S*-transferases (GST)-based metabolic resistance, are driving the insecticide resistance phenotype [8].

Two new types of dual-active ingredient ITNs were recently evaluated in clinical trials in areas with pyrethroid-resistant vectors. The epidemiological efficacy of an ITN combining pyrethroids and an insect growth regulator (pyriproxyfen) was evaluated in Burkina Faso, Tanzania and Benin [9–11]. Whilst the Olyset Duo ITN (containing permethrin + pyriproxyfen; Sumitomo Chemical) showed moderate clinical protection against malaria when compared to pyrethroid-only nets in Burkina Faso [9], the Royal Guard ITN (RG; Disease Control Technologies, Greer, SC, USA), which contains alphacypermethrin + pyriproxyfen, was not superior to standard ITNs in trials in Benin or Tanzania [10, 11]. In contrast, the Interceptor G2 ITN (IG2; BASF AGRO B.V./BASF Nederland B.V., Arnhem, The Netherlands; BASF, Ludwigshafen, Germany), containing pyrethroid + the pyrrole class pro-insecticide chlorfenapyr, showed significantly increased protective efficacy against malaria in both the Benin and Tanzania trials [4]. Consequently, the WHO has recently recommended the use of pyrethroid-chlorfenapyr-based nets against resistant malaria vectors []. Extrapolating from the results of clinical trials to predict the entomological efficacy of these next-generation ITNs in different epidemiological and ecological settings requires an understanding of the local mosquito population responses to different ITNs. Given the resources and time required for large-scale randomised controlled trials, repeating these in all settings is not practical. Malaria transmission models that were parameterised with data from semi-field studies (experimental hut trials [EHTs]) have been shown to be useful proxies for predicting the epidemiological efficacy of pyrethroid-only and pyrethroid-pyrrole ITNs [12, 13].

We performed EHTs to evaluate the performance of dual-active-ingredient ITNs compared to standard pyrethroid-only nets against pyrethroid-resistant *An. gambiae* s.l. mosquitoes from Za-Kpota, southern Benin. We also assessed the sub-lethal impacts of these new types of ITNs on the mosquitoes’ reproductive success and longevity.

## Methods

### Study site

Experimental hut trials were carried out at the Tropical Infectious Diseases Research Centre (TIDRC) field station in Za-Kpota district (7°10′58.4ʺN, 2°17′15.3ʺE), southern Benin. TIDRC is in a subtropical climate zone with two wet seasons (March-July and October–November) and two dry seasons (December-March and August–September). Monthly rainfall during the periods when the experiments were performed (July to August 2021 and October to November 2021) ranged from 1000 mm to 1200 mm (Agence pour la Sécurité et la Navigation Aérienne [ASECNA] of Benin), the mean relative humidity (RH) was 71% ± 5% and daily temperatures ranged from 29 °C to 33 °C. The main malaria vectors in the study area are pyrethroid-resistant *An. gambiae* s.l. [14].

### Insecticide susceptibility

*Anopheles gambiae* s.l. larvae and pupae were collected from breeding sites near the experimental hut station at Za-Kpota in July and September 2021 using previously described methods [15] and transported to the insectary of the Tropical Infectious Diseases Research Centre, University of Abomey-Calavi, where they were reared until the adult stage for bioassays. Mosquitoes were maintained under standard insectary conditions of 70 ± 8% RH and 27 ± 2 °C ambient temperature.

To assess the susceptibility of *An. gambiae* s.l. from Za-Kpota to the active ingredients in the ITNs to be tested, namely the Olyset Plus (permethrin + PBO), PermaNet 3.0 (deltamethrin + PBO) and Interceptor G2 (alpha-cypermethrin + chlorfenapyr) ITNs, we performed the WHO tube assay and bottle tests. WHO tube tests were carried out on 3- to 5-day-old F0 *An. gambiae* s.l. adults according to the WHO protocol [16]. In brief, mosquitoes were exposed to filter papers impregnated with 0.05% alpha-cypermethrin, 0.75% permethrin or 0.05% deltamethrin (pyrethroids). Non-impregnated filter paper was also used as the control. Mortality was recorded 24 h after exposure to each insecticide. The insecticide-treated filter papers were obtained from the WHO via the Liverpool School of Tropical Medicine (LSTM), and their quality was assessed against susceptible *An. gambiae* sensu stricto (*An. gambiae* s.s.) mosquitoes (Kisumu strain). A PBO synergist test was also performed: mosquitoes were pre-exposed to 4% PBO and then exposed to 0.05% deltamethrin [16].

Bottle bioassays were performed using the discriminating concentration of chlorfenapyr (100 µg/bottle) in accordance with the WHO protocol [16], and mortality was recorded at 72 h post-exposure. The* Anopheles* species among the *An. gambiae* s.l. complex used in the susceptibility tests were identified using *An. gambiae* species-specific PCR as either *An. gambiae* s.s., *An. coluzzii* or *An. arabiensis* [17].

### Description of treatments used in the EHTs

Two rounds of EHTs were performed (36 collection nights per round) during the long rainy season from July to August 2021 and during the short rainy season from September to November 2021. Nine different treatments were assessed over both EHTs, with untreated nets used as a negative control. To test the impact of net age, aged nets (PermaNet 3.0; Interceptor G2; Interceptor) were collected from households in the Cascades region of Burkina Faso 2 years post-distribution. All of the nets were 180 cm long × 170 cm wide × 170 high; the chemical and fabric specifications of each of the nets are described in Table 1. Prior to use in the experimental huts all nets were aired in the shade for 24 h.

**Table 1:** Specifications of insecticide-treated net products assessed in experimental hut trials

EHT number	Product name	Abbreviation	Manufacturer	Fabric type and weave	Active ingredient target doses
Trial 1	Untreated net	UTN	Bayer AG, Leverkusen, Germany	Polyester (100 denier)	No insecticide product
	Interceptor®	Interceptor	BASF AGRO B.V./BASF Nederland B.V. Arnhem (NL); BASF, Ludwigshafen, Germany	Polyester (100 denier)	Alpha-cypermethrin at 200 mg/m^2^
	Interceptor® (Aged) with 2 years of utilisation	Interceptor Aged	BASF AGRO B.V./BASF Nederland B.V. Arnhem (NL); BASF, Ludwigshafen, Germany	Polyester (100 denier)	Alpha-cypermethrin at 200 mg/m^2^
	Interceptor® G2	IG2	BASF AGRO B.V./BASF Nederland B.V. Arnhem (NL); BASF, Ludwigshafen, Germany	Polyester (100 denier)	Alpha-cypermethrin at 100 mg/m^2^ + Chlorfenapyr: 200 mg/m^2^
	Royal Guard® previously used (4 months prior to the experiment) in experimental hut and stored at 4 °C	RG Used	Disease Control Technologie, LLC, Greer, SC, USA	Polyethylene (150 denier)	Alpha-cypermethrin at 5.83 g/kg +Pyriproxyfen at 5.54 g/kg
	Royal Guard®	RG	Disease Control Technologie, LLC, Greer, SC USA	Polyethylene (150 denier)	Alpha-cypermethrin at 5.83 g/kg and Pyriproxyfen at 5.54 g/kg
Trial 2	Untreated net	UTN	Bayer AG, Leverkusen, Germany	Polyester (100 denier)	No insecticide product
	PermaNet 3.0	P3	Vestergaard Frandsen, Lausanne, Switzerland	Polyester (roof: 100 denier, sides: 75 denier)	Deltamethrin: 4.0 g/kg (roof) 2.8 g/kg (sides) PBO: 25 g/kg(roof)
	PermaNet 3.0 with 2 years of utilisation	P3 Aged	Vestergaard Frandsen, Lausanne, Switzerland	Polyester (roof: 100 denier, sides: 75 denier)	Deltamethrin: 4.0 g/kg (roof) 2.8 g/kg (sides) PBO: 25 g/kg(roof)
	PermaNet 2.0	P2	Vestergaard Frandsen, Lausanne, Switzerland	Polyester (100 denier)	Deltamethrin: 1.4 g/kg
	Interceptor® G2 (Aged) with 2 years of utilisation	IG2 Aged	BASF AGRO B.V./BASF Nederland B.V. Arnhem (NL)	Polyester (100 denier)	Alpha-cypermethrin at 100 mg/m^2^ + Chlorfenapyr at 200 mg/m^2^
	Interceptor® G2	Interceptor G2	BASF AGRO B.V./BASF Nederland B.V. Arnhem (NL); BASF, Ludwigshafen, Germany	Polyester (100 denier)	Alpha-cypermethrin at 100 mg/m^2^ + Chlorfenapyr at 200 mg/m^2^

The target dose was defined as the amount of chemical

*ETH* Experimental hut trial, *ITNs* insecticide-treated nets, NL The Netherlands, * PBO* piperonyl butoxide

### EHTs procedure

The experimental huts used for the open eaves’ experiments were typical of the West African model []. Briefly, the huts were made of concrete bricks, with a corrugated iron roof, ceilings lined with palm thatch and cement plaster on the inside. Each hut was elevated on concrete plinths surrounded by water-filled moats to prevent the entry of mosquito predators and equipped with veranda traps to capture exiting mosquitoes. Mosquitoes enter the hut through four 1-cm-wide window slits that are located on three sides of the hut. Prior to the experiment, an awareness session was held with potential study volunteers (sleepers), also to obtain their consent; only consenting volunteers were included. Treatments were allocated to the experimental huts on a weekly basis, and volunteer sleepers were rotated daily between huts using a randomised Latin square design [18]. To simulate a worn net, each net was deliberately holed with six 4 × 4-cm holes (2 holes on each side and 1 hole on each end) according to WHO protocol [18]. To attract free-flying mosquitoes, volunteer sleepers slept in the experimental huts between 20:00 hours and 05:30 hours. Each morning, alive and dead mosquitoes were collected from the different compartments inside the hut [19, 20]. All live female *An. gambiae* s.l. were given access to a 10% honey solution and delayed mortality was recorded after 72 h for all of the treatments.

Surviving blood-fed mosquitoes were placed individually in egg-laying chambers, consisting of plastic cups fitted with an untreated net and containing approximately 50 ml of dechlorinated tap water. The chambers were monitored daily for egg laying, and the number of eggs laid by each female mosquito was recorded. A pinch of larval food (TetraMin® Baby Fish Food; Tetra GmbH, Melle, Germany) was added to each chamber containing eggs, and the number of larvae hatching was recorded and monitored until adult emergence. All live mosquitoes collected in the experimental huts were monitored until death to assess their longevity. A sample of the collected female *An. gambiae* s.l. were subjected to molecular species identification. Briefly, genomic DNA of mosquitoes was extracted using the 2% cetyl trimethyl ammonium bromide (CTAB) method. Species of the *An. gambiae* complex in the sample set were identified using species-specific PCR [17].

Overall, the outcomes from the EHTs were: (i) deterrence (the proportional reduction of mosquito entry into huts with ITNs relative to huts with untreated nets; (ii) 72-h mortality rate (the number of mosquitoes dead after 72 h as a proportion of the total numbers entering the experimental huts with that treatment); (iii) exophily rate (estimated as the number of mosquitoes collected from the verandas as a proportion of all mosquitoes collected in the given experimental hut); (iv) blood-feeding rate (estimated as the proportion of mosquitoes collected that had blood fed for each experimental hut); (v) oviposition rate (estimated as the proportion of surviving blood-fed mosquitoes that laid eggs); (vi) fecundity (the mean number of laid eggs per surviving blood-fed mosquito); (vii) fertility (the mean number of emerged adults per surviving blood-fed mosquito); and (viii) longevity (number of days alive mosquitoes survive after collection from experimental huts).

### Data analysis

Data were recorded in specifically designed forms, entered into Microsoft Excel (Microsoft Corp., Redmond, WA, USA) and analysed using R statistical software version 4.3.1 [21]. Susceptibility test results were interpreted following the WHO criteria [16]. Mosquito populations were considered to be susceptible to an insecticide if the mean mortality was ≥ 98% and resistant if the mean mortality was ≤ 90% mortality. Since no mortality was recorded in controls, Abbott’s formula was not necessary to correct the mortality rates.

Outcomes from the EHTs were analysed. Deterrence was modelled using a generalised linear mixed model (GLMM) with a log link and a negative binomial distribution. Proportional data were analysed using GLMMs with a logit link and a binomial distribution (mortality 72 h) or beta-binomial distribution (exophily rate, blood-feeding rate and oviposition rate). All GLMMs were performed using the lme4 R package [22]. Fecundity and fertility were described using descriptive statistics. All models were fitted considering sleepers, huts and weeks as random effects variables while treatment was considered as a fixed effect variable. For the subsequent analysis, mosquitoes from the two trials were pooled. Taking into account the variability that could be induced by the two rounds of EHTs performed, several GLMMs were run for each parameter, systematically including "round" as a fixed effect. The final models were selected without a round included in the model according to the Akaike information criterion (AIC). In addition, we evaluated model fit by performing a quantile test, uniformity test and dispersion test using the DHARMa R package [23].

Three levels of comparison were determined: (i) untreated nets (UTNs) versus ITNs; (ii) standard net PermaNet 2.0 (P2) versus ITNs; and (iii) ITNs versus aged ITNs. The effects of treatment, physiological status and collection location on mosquito survivorship were analysed using weighted Cox regression to generate unbiased averaged hazard ratios (HR) and their corresponding 95% confidence intervals (CIs) since the proportional hazard assumption was violated [24]. This analysis was performed using the Coxphw R package with date as a cluster [25].

## Results

### Susceptibility of *Anopheles gambiae* s.l. to insecticides at Za-Kpota

Pyrethroid resistance was observed in field *An. gambiae* mosquitoes subjected to susceptibility tests. Of the 700 *An. gambiae* s.l. adult female mosquitoes exposed, 72.9% and 27.1% were identified as *An. coluzzii* and *An. gambiae* s.s., respectively, from the June 2021 collections. Regarding the adults obtaining from the September 2021 larvae collections, mosquito populations comprised 71.4% *An. coluzzii* and 28.6% *An. gambiae* s.s. Following exposure to discriminating doses, the 24-h mortality rates of *An. coluzzii* and *An. gambiae* s.s. were 17.5% and 28.3%, respectively, for permethrin, and 62.9% and 56.8%, respectively, for deltamethrin (Table 2). Twenty-four-hour mortality rates of 90.9% (*An. coluzzii*) and 93.8% (*An. gambiae s.s.)* were recorded with discriminating doses of alpha-cypermethrin. There were no significant differences in 24-h mortality between *An. gambiae* s.s. and *An. coluzzii* (*df* = 1,* χ*^2^ = 0.43, *P* = 0.508). When pre-exposure to PBO was followed by exposure to the discriminating dose of deltamethrin, the mortality rate reached 100% for both *An. coluzzii* and *An. gambiae* s.s. Both *An. coluzzii* and *An. gambiae* s.s. were susceptible to chlorfenapyr, with 72-h mortality rates of 100% and 98.07%, respectively (Table 2). All tested insecticides induced 100% mortality rate in the laboratory-maintained susceptible *An. gambiae* s.s. Kisumu strain.

**Table 2:** Insecticide susceptibility data recorded according to WHO and CDC bottle methods against field F0 female *Anopheles gambiae* sensu lato mosquitoes collected in July and September 2021

Insecticides	Susceptibility test method	Sibling species	Total* n* mosquitoes tested	Mortality rate (%)	95% CI	Resistance status
Alpha-cypermethrin (0.05%)	WHO tube	*An. coluzzii*	122	90.9	85.8–96.14	R
		*An. gambiae* s.s	48	93.8	86.6–100	R
Deltamethrin (0.05%)	WHO tube	*An. coluzzii*	116	62.9	54.01–71.8	R
		*An. gambiae* s.s.	44	56.8	41.58–72.05	R
Permethrin (0.75%)	WHO tube	*An. coluzzii*	114	17.5	10.4–24.6	R
		*An. gambiae* s.s.	46	28.3	14.74–41.78	R
PBO (4%) + Deltamethrin (0.05%)	WHO tube	*An. coluzzii*	120	100	0	/
		*An. gambiae* s.s.	40	100	0	/
Chlorfenapyr (100 µg/ml)	CDC bottle	*An. coluzzii*	48	100	0	S
		*An. gambiae* s.s.	52	98.07	94.27–100	S

Mortality was recorded 72 h following exposure for Chlorfenapyr and 24 h for the other insecticides

*CI* Confidence intervals, *R* resistant, *S* susceptible,* s.s.* sensu stricto

### Experimental hut results

#### Mosquito abundance and species identification

**Fig. 1:**
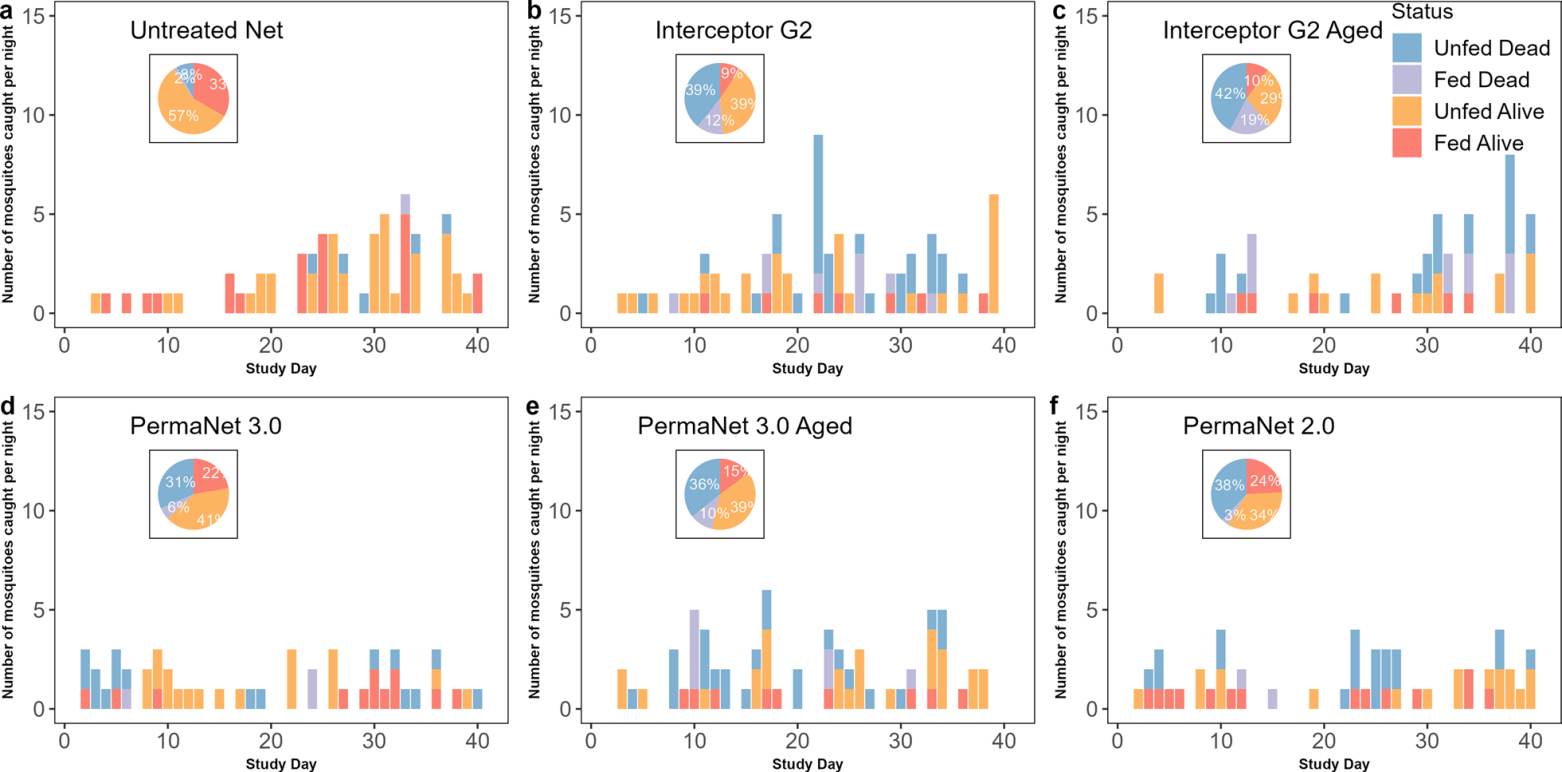
Distribution of collected *Anopheles gambiae* sensu lato mosquitoes according to their physiological status in each treatment from the experimental hut trials during the short rainy season from September to November 2021. Histograms represent the number by collection day, and pie charts represent the overall proportions. **a** Untreated net, **b** Interceptor G2 net, **c** Interceptor G2 Aged net, **d** PermaNet 3.0 net, **e** PermaNet 3.0 Aged net, **f** PermaNet 3.0 net

**Fig. 2:**
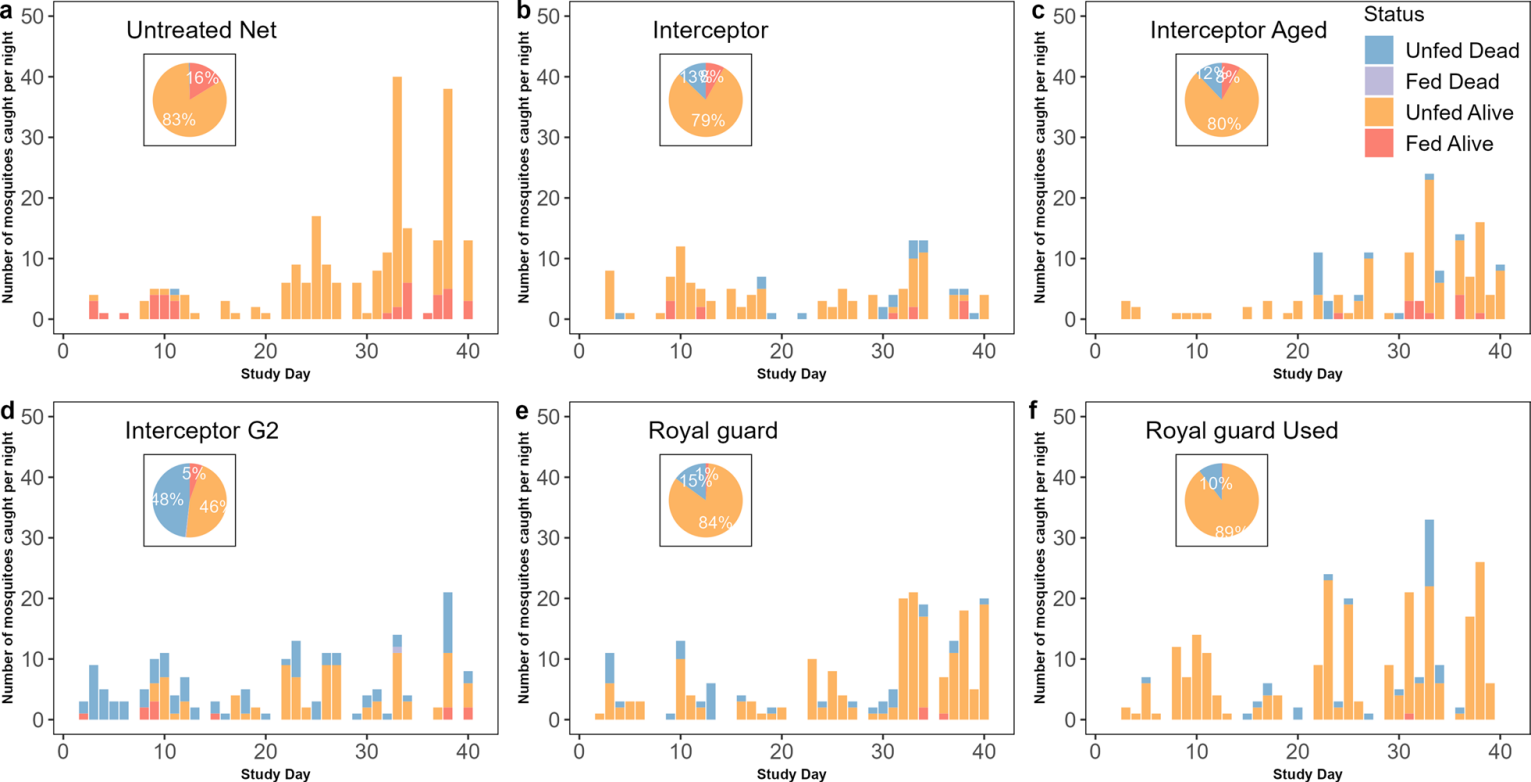
Distribution of collected *Anopheles gambiae* sensu lato mosquitoes according to their physiological status in each treatment from experimental hut trials during the long rainy season from July to August 2021. Bar charts represent the number by collection day, and pie charts represent the overall proportions. **a** Untreated net, **b** Interceptor net, **c** Interceptor Aged net, **d** Interceptor G2 net, **e** Royal Guard net, **f** Royal guard Used net

Overall, a total of 9353 mosquitoes comprising 593 *An. gambiae* s.l. (6.3%), 360* Culex* spp. (3.8%) and 8400 *Mansonia* spp. (89.81%) were collected during the short rainy season trial. For the long rainy season trial, 4959 mosquitoes comprising 1751 *An. gambiae* s.l. (35.3%), 81 *Culex* spp. (1.63%) and 3127 *Mansonia* spp. (63.1%) were collected. Of the 2344 free-flying *An. gambiae* s.l. females collected during the 72 nights of collection (36 nights for each trial round), 786 were sampled randomly from both the ETHs and identified to species level by molecular techniques. The predominant species was *An. gambiae* s.s. (99.4%). Overall, the collected *An. gambiae* s.s. mosquitoes were mostly found as unfed mosquitoes (61.4%) (Figs. 1, 2).

#### *Anopheles gambiae* s.s. entry into and exiting from huts

None of the ITNs tested significantly deterred mosquito entry into the huts relative to the UTN (Table 3). A significantly higher exophily was recorded with the Interceptor G2 net (IG2) (odds ratio [OR] = 2.1, *P* < 0.0013) and Royal Guard net (OR = 1.6, *P* < 0.04) compared to that of the UTN (Table 4).

**Table 3:** Comparative odds ratio of deterrence by contrasting treatments

Treatments	ITN vs untreated net		ITN vs PermaNet 2.0 Unwashed		ITN Aged vs ITN Unwashed	
	Odds ratio (95% CI)	*P*-value	Odds ratio (95% CI)	*P*-value	Odds ratio (95% CI)	*P*-value
Untreated net	–	–	–	–	–	–
PermaNet 2.0	0.48 (0.13–1.74)	0.52	–	–	–	–
Interceptor	0.71 (0.44–1.16)	0.30	1.48 (0.40–5.44)	0.91	–	–
Interceptor Aged	0.84 (0.52–1.35)	0.22	1.74 (0.47–6.40)	0.75	1.18 (0.72–1.94)	0.88
PermaNet 3.0	0.40 (0.11–1.46)	0.29	0.83 (0.15–4.48)	0.99	–	–
PermaNet 3.0 Aged	0.63 (0.18–2.27)	0.84	1.31 (0.25–6.97)	0.98	1.57 (0.29–8.41)	0.93
Interceptor G2	1.02 (0.65–1.61)	0.99	2.12 (0.59–7.64)	0.49	–	–
Interceptor G2 Aged	0.53 (0.15–1.90)	0.63	1.09 (0.21–5.84)	0.99	0.52 (0.14–1.86)	0.60
Royal Guard	1.47 (0.99–2.19)	0.05	3.04 (0.85–10.88)	0.11	–	–

*CI* Confidence interval, ITNs insecticide-treated nets

**Table 4:** Comparative odds ratio of exophily rate by contrasting treatments

Treatments	ITN vs untreated net		ITN vs PermaNet 2.0 Unwashed		ITN Aged vs ITN Unwashed	
	Odds ratio (95% CI)	*P-*value	Odds ratio (95% CI)	*P*-value	Odds ratio (95% CI)	*P*-value
Untreated net	–	–	–	–	–	–
PermaNet 2.0	1.31 (0.59–2.87)	0.86	–	–	–	–
Interceptor	1.22 (0.64–2.32)	0.90	0.93 (0.39–2.21)	0.99	–	–
Interceptor Aged	1.66 (0.91–3.03)	0.13	1.27 (0.55–2.91)	0.92	1.36 (0.68–2.73)	0.72
PermaNet 3.0	0.81 (0.36–1.79)	0.93	0.62 (0.23–1.64)	0.64	–	–
PermaNet 3.0 Aged	1.09 (0.54–2.17)	0.99	0.83 (0.34–2.04)	0.97	1.35 (0.54–3.34)	0.88
Interceptor G2	2.08 (1.24–3.47)	0.001***	1.59 (0.73–3.43)	0.46	–	–
Interceptor G2 Aged	1.10 (0.51–2.36)	0.99	0.84 (0.33–2.18)	0.98	0.53 (0.25–1.12)	0.13
Royal Guard	1.60 (1.00–2.56)	0.04	1.22 (0.58–2.57)	0.93	–	–

*CI* Confidence interval, ITN insecticide-treated net

*Significant difference at *P* < 0.005

#### Blood-feeding rates of wild* An. gambiae* s.s.

The proportion of blood-fed *An. gambiae* s.s. in the experimental huts with UTNs was 14.6%, compared to 0.5–19.5% blood-fed *An. gambiae* s.s. in the experimental huts with ITNs (Table 5). The proportion of blood-fed *An. gambiae* s.s. decreased significantly in the huts equipped with Royal Guard (RG; OR = 0.05, *P* < 0.001) nets compared to those equipped huts with UTN or the standard PermaNet 2.0 net (P2) (OR = 0.07, *P* = 0.0021). No significant inhibition of blood feeding was observed in huts equipped with Interceptor, Interceptor Aged, Interceptor G2 (IG2), Interceptor G2 Aged (IG2 Aged), PermaNet 3.0 (P3) and PermaNet 3.0 Aged (P3 Aged) nets compared those with UTNs or the standard net P2. There was no significant difference in blood feeding between huts with ITNs and those with aged ITNs in any pairwise comparison (Table 5).

**Table 5:** Blood-feeding rate by treatment and comparative odds ratio by contrasting treatments

Treatments	Blood-feeding rate (%)	ITN vs untreated nets		ITN vs PermaNet 2.0 Unwashed		ITN Aged vs ITN Unwashed	
		Odds ratio (95% IC)	*P*-value	Odds ratio (95% IC)	*P*-value	Odds ratio (95% IC)	*P*-value
Untreated net	14.63	–	–	–	–		
PermaNet 2.0	17.58	0.70 (0.24–2.05)	0.88	–	–	–	–
Interceptor	6.41	0.39 (0.10–1.52)	0.31	0.55 (0.11–2.91)	0.85	–	–
Interceptor Aged	5.53	0.38 (0.10–1.36)	0.22	0.54 (0.11–2.63)	0.80	0.97 (0.19–4.89)	1
PermaNet 3.0	19.48	0.77 (0.25–2.34)	0.95	1.09 (0.35–3.45)	0.99	–	–
PermaNet 3.0 Aged	13.93	0.45 (0.14–1.39)	0.29	0.64 (0.20–2.06)	0.81	0.58 (0.17–1.96)	0.72
Interceptor G2	7.91	0.45 (0.19–1.08)	0.089	0.64 (0.21–1.96)	0.79	–	–
Interceptor G2 Aged	15.74	0.51 (0.16–1.65)	0.50	0.72 (0.21–2.45)	0.93	1.12 (0.33–3.79)	0.9981
Royal Guard	0.52	0.05 (0.01–0.27)	< 0.0010***	0.07 (0.01–0.49)	0.0021**	–	–

*CI* Confidence interval, ITN insecticide-treated net

*Significant difference at* P* < 0.005; **significant difference at* P* < 0.001

#### Seventy-two-hour mortality rates of wild* An. gambiae* s.s.

The 72-h mortality of *An. gambiae* s.s. was 44.3% in the experimental huts with UTNs and ranged from 56.4% to 90.1% in the experimental huts with ITNs. Among the ITNs, the lowest 72-h mortality rate occurred in huts equipped with the standard P2 net (56.04%) and Interceptor net (59.41%) (Table 6). The highest killing effect was recorded in the hut with the IG2 net (OR = 8.9, *P* < 0.001) compared to the hut with UTNs (Table 6). Similarly, the IG2 net (OR = 5.9, *P* < 0.001) induced the higher mortality rate when compared to the P2 net. Mortality was also higher in the huts with the P3 Aged net (OR = 2.5, *P* = 0.01) and RG net (OR = 2.4, *P* = 0.0011) compared those equipped with the P2 net (Table 6). Surprisingly, the Interceptor Aged net (OR = 2.25, *P* = 0.011) induced significantly higher mortality than the Interceptor net; conversely, the IG2 Aged net (OR = 0.38, *P* = 0.006) induced significantly lower mortality than the IG2 net.

**Table 6:** The 72-h mortality rate by treatment and comparative odds ratio by contrasting treatments

Treatments	72-h Mortality rate (%)	ITN vs untreated net		ITN vs PermaNet 2.0 Unwashed		ITNs Aged vs ITN Unwashed	
		Odds ratio (95% CI)	*P*-value	Odds ratio (95% CI)	*P*-value	Odds ratio (95% CI)	*P*-value
Untreated net	44.36	–	–	–	–	–	–
PermaNet 2.0	56.04	1.52 (0.80–2.88)	0.38	–	–	–	–
Interceptor	59.41	1.25 (0.67–2.32)	0.84	0.82 (0.38–1.78)	0.94	–	–
Interceptor Aged	69.79	2.82 (1.64–4.83)	< 0.0010***	1.86 (0.91–3.80)	0.12	2.25 (1.13–4.49)	0.011*
PermaNet 3.0	74.04	3.17 (1.49–6.78)	< 0.0010***	2.09 (0.84–5.19)	0.17	–	–
PermaNet 3.0 Aged	75.41	3.84 (2.03–7.27)	< 0.0010***	2.53 (1.13–5.68)	0.01	1.21 (0.49–3.00)	0.97
Interceptor G2	90.11	8.95 (5.04–15.91)	< 0.0010***	5.90 (2.75–12.64)	< 0.0010***	–	–
Interceptor G2 Aged	75.93	3.36 (1.70–6.64)	< 0.0010***	2.21 (0.96–5.13)	0.072	0.38 (0.17–0.82)	0.006**
Royal Guard	75.71	3.66 (2.50–5.38)	< 0.0010***	2.41 (1.30–4.48)	0.001	–	–

*CI* Confidence interval, ITN insecticide-treated net

*Significant difference at* P* < 0.05; **significant difference at* P *< 0.001

#### Effect of exposure to the ITNs on oviposition, fecundity, and fertility

Only a low number of blood-fed mosquitoes were collected from EHTs and available for follow-up analysis (*n* = 60 for UTN and *n* = 105 for all ITNs combined). Oviposition was reduced in mosquitoes collected from huts with ITNs (of any type) compared to the control hut with UTNs (Table 7). No significant difference was observed between oviposition rates in huts with the standard P2 net and the other ITNs (Table 7). Generally, the mean number of eggs laid (fecundity) and the number of adults that emerged (fertility) from surviving blood-fed *An. gambiae* s.s. mosquitoes were lower in the huts with ITNs than in those with UTNs (Table 7). The mean number of eggs laid per female was 44.8 (95% CI 37.7–51.8) for *An. gambiae* s.s. mosquitoes collected from huts with UTNs. Lower mean numbers of eggs laid were observed for mosquitoes collected from huts with the IG2 (33.3; 95% CI 21.4–45.8]), P3 (33.4; 95% CI 27.3–39.4) and IG2 Aged (28.8; 95% CI 18.2–39.4]) nets. The mean number of *An. gambiae* s.s. adults that emerged was 44.7 (95% CI 37.6–51.8) in mosquitoes collected in huts with UTNs, with lower mean numbers of adults emerged for huts equipped with P3 (23.9; 95% CI 18.0–29.8) and Interceptor (18.3; 95% CI 5.6–31.1) nets (Table 7).

**Table 7:** Oviposition, fecundity and fertility in blood-fed female *Anopheles gambiae* collected from the experimental hut trials

Treatments	Total no. of mosquitoes blood fed	Oviposition rate (%) (95% CI)	Number of surviving blood-fed mosquitoes	Fecundity		Fertility	
				Total no. of eggs laid	Number eggs/no. of females (95% CI)	Total no. of emerged adults	Number of emerged adults/no. of females (95% CI)
Untreated net	60	91.7 (84.5–98.9)	55	2463	44.8 (37.7–51.8)	2457	44.7 (37.6–51.8)
PermaNet 2.0	16	43.8 16.4–71.1)	7	262	37.4 (28.9–45.9)	185	26.4 (20–32.9)
Interceptor	8	37.5 (5.5–80.3)	3	130	43.3 (− 27–114)	55	18.3 (5.59–31.1)
Interceptor Aged	12	50.2 (16.8–83.2)	6	246	41 (25.1–56.9)	177	29.5 (22.9–36.1)
Interceptor G2	18	33.3 (9.2–57.5)	6	200	33.3 (21.4–45.3)	188	31.3 (23.4–39.3)
Interceptor G2 Aged	16	100	16	461	28.8 (18.2–39.4)	453	28.3 (17.6–39)
Royal Guard	3	0	0	0	NA	NA	NA
PermaNet 3.0	15	53.3 (24.7–81.9)	8	267	33.4 (27.3–39.4)	191	23.9 (18–29.8)
PermaNet 3.0 Aged	17	11.8 (5.3–28.8)	2	47	23.5 (− 123–170)	41	20.5 (− 138–179)

*CI* Confidence intervals, *NA* not applicable

#### Effect of exposure to the ITNs on longevity

**Fig. 3:**
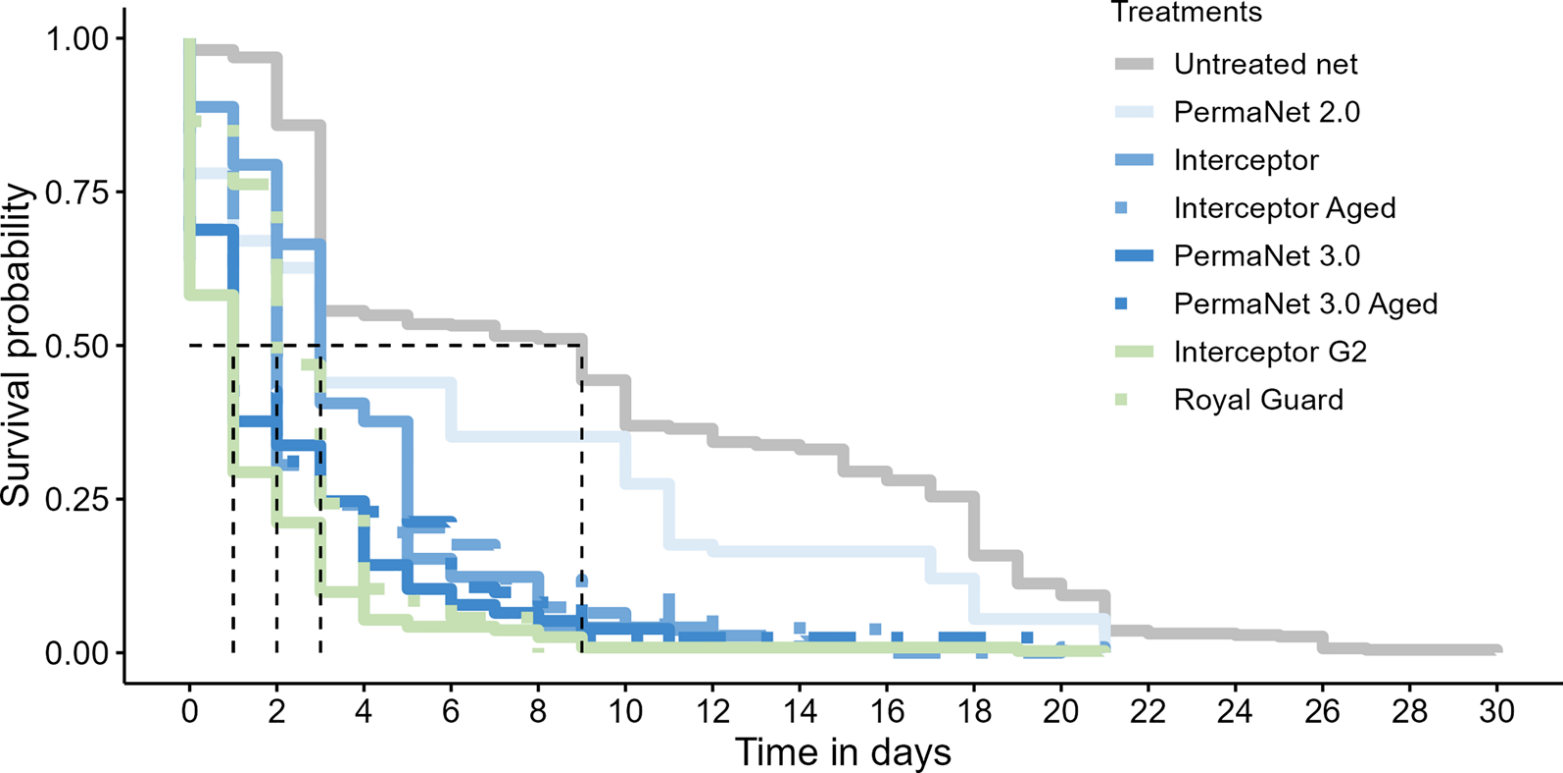
Kaplan–Meier curves describing survival rates as a function of time in *Anopheles gambiae* sensu lato mosquitoes collected in experimental hut trials. Vertical dotted lines indicate median survival time. Day 0 corresponds to mosquito collection days in experimental huts. P2, P3, PermaNet 2.0, 3.0, respectively; IG2, Interceptor G2; RG, Royal Guard; UTN, untreated net; see also Table 1

**Fig. 4:**
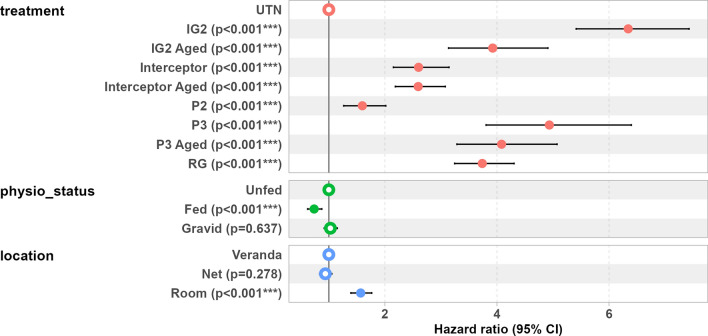
Comparative hazard ratio of death according to the treatments, collection location and physiological status in *Anopheles gambiae.* Error bar represents the 95% confidence interval, P2, P3, PermaNet 2.0, 3.0, respectively; IG2, Interceptor G2; RG, Royal Guard; UTN, untreated net; see also Table 1

A strong negative effect was observed on the longevity of *An. gambiae* s.s. after exposure to all of the ITNs. The median survival time of female *An. gambiae* s.s. mosquitoes collected from huts with UTNs was 9 days, and varied between 1 and 3 days for *An. gambiae* s.s. exposed to ITNs (Fig. 3). A significantly higher risk of death was observed with all ITNs compared to UTN (Fig. 4). The highest risk was recorded in *An. gambiae* s.s. exposed to the IG2 (HR = 6.5, 95% CI 5.6–7.6, *P* < 0.001) and P3 (HR = 4.4, 95% CI 3.4–5.7, *P* < 0.001) nets (Fig. 4). Compared to mosquitoes collected in the veranda, the risk of death was significantly higher in those collected in the room (HR = 1.8, 95% CI 1.6–1.9, *P* < 0.001). The risk of death was also higher in gravid mosquitoes (HR = 1.1, 95% CI 1–1.2, *P* = 0.04) compared to unfed mosquitoes. The risk of death was significantly lower in fed mosquitoes (HR = 0.73, 95% CI 0.6–8.9, *P* < 0.001) than in unfed mosquitoes (Fig. 4).

## Discussion

African malaria vectors populations display a high level of pyrethroid resistance [3]. Country-wide surveys over the last decade in Benin have reported that pyrethroid resistance is widespread in malaria vector populations [26, 27]. Investigating tools that can complement or replace existing ones are therefore necessary to strengthen resistance management plans [28]. This study provides key information on the performance of dual-active-ingredient ITNs (Interceptor G2, PermaNet 3.0 and Royal Guard) and their sub-lethal impacts against a pyrethroid-resistant population of *An. gambiae* s.s. from Za-Kpota in southern Benin using experimental hut trials.

A low mortality rate to pyrethroid insecticides (permethrin and deltamethrin) was recorded for *An. gambiae* s.s. mosquitoes collected from Za-Kpota using the WHO susceptibility test. This result confirms the presence of resistance to those insecticides, which has also been reported in the neighbouring localities of Za-Kpota in southern Benin [26, 27]. A synergist assay with 4% PBO and deltamethrin was carried out in the Za-Kpota mosquito population, with the results revealing a potential involvement of cytochrome P450 genes in observed phenotypic resistance. This indicates that the pyrethroid resistance observed might also be driven by metabolic resistance mechanisms in the *An. gambiae* s.s. population of Za-Kpota.

The 72-h mortality rates were considered in the EHTs. The pyrethroid-PBO-based net (PermaNet 3.0 [P3]) displayed higher toxicity against field free-flying pyrethroid-resistant *An. gambiae* s.s. when compared to the pyrethroid-only based net (PermaNet 2.0 [P2]). The PermaNet 3.0 net is treated with a high concentration of deltamethrin on the side panels (2.1 g/kg) and with 4.0 g/kg deltamethrin + PBO (25 g/kg) on the roof panel. Thus, the high toxicity observed with PermaNet 3.0 could be due to the amount of active ingredients on this net. Given the well-known synergistic effect of PBO on pyrethroid resistance [29] and the data from the WHO susceptibility test, the difference in the killing effect recorded may be due to the partial or complete restoration of susceptibility to deltamethrin, thus increasing the efficacy of PermaNet 3.0 over PermaNet 2.0. The same trend was observed between Olyset and Olyset Plus [30], confirming the improved protective role of PBO nets on malaria prevalence in areas where the resistance phenotype is mainly driven by cytochrome P450 genes [8–12].

Nevertheless, PermaNet 3.0 does not provide total personal protection (induced mortality of < 80%) [29, 31]. These results highlight the possible presence of resistance mechanisms other than metabolic resistance mediated by monooxygenases in the vector population. They also highlight concerns regarding the efficacy of PermaNet 3.0, which have been reported in several previous studies in southern Benin [32, 33], raising questions regarding the true performance of PermaNet 3.0 against resistant malaria vectors where mechanisms such as GST-based metabolic resistance occur [8, 34, 35]. Such concerns have led to more attention being paid to the new dual-active-ingredient ITNs (Interceptor G2 [IG2] and Royal Guard [RG]), each of which contain two products with different modes of action. Interestingly, in the present study, a higher mortality rate was recorded with Interceptor G2 and Royal Guard nets against pyrethroid-resistant *An. gambiae* s.s. mosquitoes than with the standard pyrethroid-only net PermaNet 2.0.

The highest mortality was observed with the Interceptor G2 net, possible due to the combined toxic effects of alpha-cypermethrin and chlorfenapyr. Chlorfenapyr is activated when the* N*-ethoxymethyl group is removed by oxidation mediated by some cytochrome P450 enzymes, producing the toxic metabolite tralopyril [36]. Tralopyril disrupts the proton gradient across mitochondrial membranes and impairs ATP production (oxidative phosphorylation), leading to cell death [37, 38]. As demonstrated in this study, cytochrome P450 enzymes are involved in pyrethroid resistance in the *An. gambiae* s.s. population at Za-Kpota. Thus, overexpression of these enzymes may enhance chlorfenapyr activation [39–41], possibly resulting in increased toxicity of nets with insecticide combinations of pyrethroids and chlorfenapyr. A similar observation has been pointed out in several experimental hut trials [42–44].

This study also showed that while the Interceptor G2 Aged (IG2 Aged) net induced a high mortality compared to the PermaNet 2.0, it induced significantly lower mortality compared to the Interceptor G2 net (IG2). This could be due to a decrease in the insecticide content of ITNs in operational use over time, as has been demonstrated in several studies [45, 46]. In contrast, Martin et al. [47] demonstrated that after 20 washes (supposed to have mimicked a 36-month-old field net), the Interceptor G2 washed net did not induce a significantly different mortality from the Interceptor G2 unwashed net, confirming the wash resistance of this net. This contrast reveals that the decrease in insecticide content of insecticide-treated nets over time would not be proportional to its reduction after washing. Future new net durability studies should focus more on aged nets, which more accurately capture ageing in the field due to different environmental conditions (temperature, humidity).

In addition to the high toxicity displayed by the three dual-active-ingredient ITNs (Interceptor G2, Royal Guard and PermaNet 3.0), these nets also had irritant properties that enabled them to inhibit blood feeding by pyrethroid-resistant *An. gambiae* s.s. mosquitoes from Za-Kpota. However, only the Royal Guard net significantly reduced the blood feeding compared to the standard net PermaNet 2.0. Of the ITNs evaluated in this study, the Royal Guard net was treated with the highest concentration of alpha-cypermethrin (216 mg/m^2^). The significant reduction in blood feeding induced by the Royal Guard net could be due to its high alphacypermethrin concentration. The latter could trigger an avoidance behaviour in the vectors [48], affecting their ability to take a blood meal. As a key parameter influencing malaria transmission potential [49], blood-feeding behaviour observed with new nets could benefit malaria vector control interventions [42–44]. This result confirms that high-dose pyrethroids would continue to play a valuable role in blood-feeding inhibition and personal protection [50].

In addition to the standard parameters such as mortality and blood feeding, the sublethal exposure effect could provide complementary information to better appreciate the performance of vector control strategies. These could include reductions in longevity, development rates, feeding, oviposition, fertility, fecundity and changes in sex ratio or behaviour [51]. Several studies have shown that insecticide resistance mechanisms can negatively affect the reproductive fitness and longevity of *Anopheles* mosquitoes [52–54]. In the present study, we analysed the sub-lethal impact of ITN exposure in collected *An. gambiae* s.s. mosquitoes using oviposition, fecundity, fertility and longevity in which a risk of death has been estimated. We found no significant difference in oviposition rate in collected blood-fed *An. gambiae* s.s. between standard net PermaNet 2.0 and the other ITNs.

Exposure to the dual-active-ingredient nets (Interceptor G2, Interceptor G2 Aged, PermaNet 3.0 and PermaNet 3.0 Aged) reduced fecundity compared to exposure to the standard net PermaNet 2.0. This reduced fecundity may be due to the synergistic action of alphacypermethrin, PBO or chlorfenapyr on fecundity in resistant mosquitoes, possibly as a result of a resource-based trade-off between fecundity and survival [55]. When mosquitoes are exposed to dual-active-ingredient nets, overuse of energy for survival could impact resource availability for fecundity. Such regulation could indicate a high cost of adaptation linked to insecticide resistance [56] which has been described in previous studies [57, 58]. The reduction in fecundity could translate, from an epidemiological point of view, into a decrease in vector density and, hence, a reduction in transmission [59].

In addition, reducing vector longevity is one of the objectives of current insecticide-based malaria control programmes and insecticide resistance management strategies. In this study, the risk of death was high with PermaNet 3.0, Interceptor G2 and Royal Guard nets, with the highest risk of death recorded with the Interceptor G2 net. This result shows once more the efficacy of these nets and, in particular, the combined toxic effects of alpha-cypermethrin and chlorfenapyr. Chlorfenapyr has a delayed impact [60] due to its mode of activation, so it could persist for several days after exposure, thus disrupting several life-history traits of the mosquito, including longevity. The present findings indicate that new nets in the community are likely to impede the developmental cycle of vectors and thus contribute to reducing malaria transmission. These sub-lethal data generated in this study, when added to available data, will undoubtedly contribute to the improvement of parameterised malaria transmission models to predict the long-term efficacy of new ITNs.

## Conclusions

Novel, suitable alternative insecticides that can complement pyrethroids and improve control of pyrethroid-resistant malaria vectors are urgently needed to maintain ITNs as a means of malaria control. The dual-active-ingredient nets Royal Guard and Interceptor G2 have shown improved entomological efficacy compared to the standard net PermaNet 2.0 and appear promising for effectively controlling insecticide-resistant malaria vectors. This study provides entomological evidence of the efficacy of these new control tools in a region of southern Benin and suggests that they can be used in this area to control malaria-resistant vectors.

## Data Availability

The datasets supporting the conclusions of this articles are available in the Figshare repository and provided via 10.6084/m9.figshare.26014042.v2.

## Abbreviations

ETH: Experimental hut trial
GST: Glutathione-*S*-transferases
ITNs: Insecticide-treated nets
PBO: Piperonyl butoxide
TIDRC: Tropical Infectious Diseases Research Centre
